# Effect of Oral Exercise on Trismus after Oral Cancer Radiotherapy: A Quasi-Experimental Study

**DOI:** 10.3390/biomedicines10112951

**Published:** 2022-11-17

**Authors:** Tsae-Jyy Wang, Kuo-Feng Wu, Hung-Ming Wang, Shu-Yuan Liang, Ting-Ru Lin, Yi-Wei Chen

**Affiliations:** 1 School of Nursing, National Taipei University of Nursing and Health Sciences, Taipei 112, Taiwan; 2 Department of Nurse-Midwifery and Women Health, National Taipei University of Nursing and Health Science, Taipei 112, Taiwan; 3 Department of Hematology/Oncology, Chang Gung Memorial Hospital at Linkou and Chang Gung University, Taoyuan City 333, Taiwan; 4 Department of Nursing, Cardinal Tien College of Healthcare and Management, New Taipei City 231, Taiwan; 5 Department of Nursing, Hualien Tzu Chi Hospital, Buddhist Tzu Chi Medical Foundation, Hualien County 970, Taiwan

**Keywords:** oral cancer, oral exercise, trismus, radiotherapy, maximum inter-incisal opening

## Abstract

Trismus is a severe complication of oral cancer treatment. Oral exercise is a potentially helpful approach for preventing or improving trismus. The study aimed to test the efficacy of an oral exercise for enhancing the maximum inter-incisal opening (MIO) in patients undergoing surgery and radiotherapy for oral cancer. This is a quasi-experimental study. A sample of 69 oral cancer patients completed the study, with 35 in the control group and 34 in the intervention group. Intervention subjects were asked to perform three 20-min oral exercise sessions per day for six months. Data on oral exercise practicing time, MIO, and mandibular function impairment were collected at the last radiotherapy exposure (T1), three months (T2), and six months (T3) after the radiotherapy. At T3, the intervention group exercised 217.1 min (95%CI: 107.4~326.7) more than the control group. The generalized estimation equations showed a statistically significant group-by-time interaction in MIO. The change in MIO score from T1 to T3, as indicated by the regression slope, was 2.5 mm (95%CI: 0.4~4.6) greater in the intervention group than in the control group. The results support the efficacy of the study intervention for improving patient exercise adherence and MIO.

## 1. Introduction

Oral cancer is an umbrella term for malignant mouth tumors, including the buccal mucosa, the front two-thirds of the tongue, the hard palate, the floor of the mouth, the lips, or the gums [1,2]. In Taiwan, oral cancer is the fifth most prevalent form of cancer [3], with a 5-year survival rate between 85% (Stage 1) and 37.3% (Stage 4) [4]. Advancements in cancer treatment have greatly improved patients’ survival rates. Surgical resection of tumors and radiotherapy (RT) are the standard treatments for oral cavity cancer [5]. However, when a larger area is removed, it may change the patient’s ability to chew, swallow, or speak. In this case, flap reconstruction surgery is needed to rebuild the tissue in the mouth, and rehabilitation exercises are performed to help the damaged tissue regain function [6]. Postoperative scarring and radiation-induced pterygia or masseter and temporomandibular joint (TMJ) fibrosis often lead to difficulty opening the mouth or trismus [7,8,9]. In addition, the size and location of the tumor and the type of resection and reconstruction surgeries can also affect the degree of restricted mouth opening. For example, patients who underwent surgery for stage I and II cancers had less destructive surgery on the primary tumor and a more conservative lymphadenectomy than patients with advanced cancer.

Trismus is generally defined as a maximal interincisal opening (MIO) smaller than 35 mm [10,11]. The incidence of trismus after head and neck cancer (HNC) treatment is between 31% and 87% [8,12,13]. Patients may begin to have trouble opening their mouths during treatment. These symptoms typically peak at six months after RT and last several years [7,14,15]. Trismus affects patients’ ability to chew and talk, severely impacting their quality of life. In addition, trismus makes it difficult to maintain oral hygiene, increasing the risk of tooth decay and periodontitis [16].

Oral exercise involving actively or passively stretching the jaw using jaw motion rehabilitation systems (e.g., TheraBite, Dynasplin, or tongue depressors) is a potentially helpful approach for preventing or improving trismus. Findings from previous studies [13,17,18] provide preliminary support for the efficacy of oral exercise in improving MIO in patients with trismus caused by HNC treatment. However, the sample sizes of the previous studies were small, and there was a high degree of heterogeneity in the oral exercises employed in these studies [18]. The ideal exercise regimen could not be determined. Additionally, the researchers included patients with different forms and stages of HNC [13], which prevented the findings from indicating the effects of oral exercise on patients with specific HNC. Most of the studies failed to provide records of exercise times. Recording exercise times would allow for dose-effect analysis and determining the ideal frequency and duration of oral exercises.

Therefore, this study aimed to test the efficacy of an oral exercise intervention in enhancing exercise practice and mouth opening in patients undergoing tumor resection and flap reconstruction surgery, as well as RT or concurrent chemoradiotherapy (CCRT) for oral cancer. We hypothesized that, over time, the intervention group would show more significant improvements in (1) mouth-opening exercise practicing time, (2) MIO (the primary outcome), and (3) mandibular function impairment compared to the control group.

## 2. Materials and Methods

### 2.1. Study Design

This is a quasi-experimental study. The study was approved by the institutional review board of the hospital, where the subjects were recruited (IRB number: 103-1668C) and registered with ClinicalTrials.gov (number: NCT05227807).

### 2.2. Subject Recruitment

A convenience sample of oral cancer patients who met the following eligibility criteria was recruited from a general hospital in Taiwan. The inclusion criteria consisted of: (1) being over the age of 18; (2) coherent; (3) speaking Mandarin or Taiwanese; (4) being diagnosed with primary oral cavity cancer by a physician (including cancer of the gums, the floor of the mouth, inner cheek, and jaw, and retromolar cancer); (5) received tumor resection surgery (including lymphatic clearance) and flap reconstruction; and (6) received RT or CCRT. Exclusion criteria consisted of: (1) inability to engage in oral exercises due to poor recovery of postoperative wounds; (2) inability to measure mouth opening distance due to the absence of incisors; and (3) HNC irrelevant to trismus (i.e., lip cancer and tongue cancer). During the first year of the study, eligible patients were consecutively recruited into the control group. During the second year of the study, patients who met the inclusion criteria were consecutively recruited to the intervention group.

The required sample size was calculated using G-Power 3.1.2. [19]. The intervention was assumed to have a moderate impact on maximum mouth opening. The f^2^-value was 0.30, the α-value was 0.05, and the desired power was 0.80. We found that a sample size of 56 subjects would be suitable, with 28 in each group. A sample of 69 oral cancer patients completed the study, with 35 in the control group and 34 in the intervention group.

### 2.3. Intervention

Intervention subjects received the oral exercise intervention immediately after their last RT exposure. The intervention consisted of 30 min of personal training, a multimedia oral exercise video, a printed educational brochure, and three follow-up calls. The subjects were asked to perform three 20-min oral exercise sessions per day for six months. They also received three follow-up calls from the interventionist in the first week, the first month, and the third month after discharge to address related issues and enhance exercise adherence.

The exercise comprises seven parts: cheek massage, jaw stretch, facial muscle movement, tongue movement, sucking, chewing, and intensive mouth opening. Delivering a message to the cheek at the beginning of the intervention relaxes the muscles and ligaments around the cheek and promotes temporomandibular joint movement. Stretching the jaw and facial muscles increases muscle-tendon elasticity, joint range of motion, and cheek control. Tongue movement and sucking exercises can prevent or improve slurred speech, eating problems, and salivation. Finally, chewing and intensive mouth-opening exercises strengthen the masticatory muscles and prevent trismus. The subjects performed the entire intervention three times a day. See Appendix A for details. The printed booklet of the exercises in Appendix A was distributed to the control group patients without further explanation.

### 2.4. Data Collection and Instruments

Data on oral exercise practicing time, MIO, and mandibular function impairment were collected in both groups at the last RT exposure (T1), three months (T2), and six months (T3) after the completion of RT. One researcher collected data in a quiet room in the clinic. The data collector measured the subjects’ MIO using the TheraBite Range-of-Motion Scale (TheraBite Corporation, West Chester, PA, USA). The subjects were asked to sit on a chair and open their mouths as wide as possible. The researcher used the range-of-motion scale to measure the vertical distance between the lower end of the upper central incisors and the upper end of the lower central incisors. A measurement of 35 mm or less was considered to represent difficulty in opening the mouth, with smaller measurements denoting more severe conditions [8,10].

The Mandibular Function Impairment Questionnaire [20] was used to assess the limitation of oral function due to mandibular function impairment. Subjects completed this self-reported questionnaire that comprised 17 items encompassing the difficulty of chewing hard and soft foods, taking large bites, laughing, eating certain foods, engaging in social activity, and speaking. The items were scored on a 5-point Likert scale (0–4). The overall score was calculated by dividing the sum of the item scores by 68, with a possible range of 0 to 1. A higher score denoted a more serious functional impairment. An overall score of ≤0.3 denoted a low degree, 0.3 ≤ 0.6 denoted a moderate degree, and >0.6 denoted a high degree of functional impairment. The scale achieved test-retest reliability of 0.80 and a Cronbach’s α between 0.63 and 0.95 when administered to patients with temporomandibular joint diseases (*n* = 95) [20]. The Chinese version of the questionnaire achieved an expert content validity index (CVI) of 0.8 and a Cronbach’s α of 0.96 when administered to oral cancer patients [21]. The present study achieved a Cronbach’s α value of 0.96.

The subjects were asked to keep an oral exercise journal and record the progress of their oral exercises each week. They were also asked to fill out a demographics questionnaire to disclose their age, gender, level of education, marital status, and occupation. We transcribed the subjects’ disease characteristics from their medical records, recording their diagnosis, cancer stage, surgical procedure, and adjuvant treatment information.

### 2.5. Statistical Analysis

Statistical analysis was performed by the Statistical Package for the Social Sciences (SPSS) v. 20 (IBM, Armonk, NY, USA). Chi-squared tests and independent-sample *t*-tests were performed to examine the homogeneity of the subjects’ demographics and disease characteristics. Chi-squared tests were used to compare the group differences in the number of subjects who continued exercise at different points. Generalized estimation equations (GEE) were used to analyze the intervention’s effect on MIO, and mandibular function impairment. Independent *t*-tests were used to analyze the group differences for the outcome variables measured at three points.

## 3. Results

### 3.1. Subject Demographics

We adopted a quasi-experimental research design. In the first stage of this study, 68 patients were approached for the control group. Six patients did not meet the eligibility criteria and were excluded (two were diagnosed with tongue cancer, three had recurrent oral cavity cancer, and one suffered from a mild postoperative stroke), and 25 refused to participate due to physical discomfort, poor mood, or disinterest. Thirty-seven eligible subjects were recruited for the control group. However, two control subjects withdrew during the research period due to physical discomfort. Therefore, a total of 35 control subjects completed this study.

In the second stage, a total of 71 patients were approached. Six patients did not meet the eligibility criteria and were excluded (one was diagnosed with tongue cancer, three had recurrent oral cavity cancer, one developed post-treatment flap atrophy and refused flap reconstruction surgery, and one had non-primary oral cavity cancer), and 28 refused to participate due to physical discomfort, poor mood, or disinterest. Thirty-seven eligible subjects were recruited for the intervention group. However, three intervention subjects withdrew during the research period due to physical discomfort. Therefore, a total of 34 intervention subjects completed this study.

Thus, 69 subjects who completed the study were included in the analysis, with 35 in the control group and 34 in the intervention group. The average age of the subjects was 50.2 (SD = 7.4). The majority were male (*n* = 66, 95.7%), and the most common cancer was buccal mucosa cancer (*n* = 43, 62.3%), which was most commonly stage IV (*n* = 36, 52.9%). All subjects received tumor excision surgeries, and 65 subjects (94.2%) also received free flap reconstructions. Forty-eight subjects (69.6%) received CCRT, and 21 (30.4%) received RT only. All RTs were performed with intensity-modulated radiation therapy (IMRT) techniques. Demographics and disease profiles were equivalent between groups at baseline (Table 1).

### 3.2. Time of Practicing Oral Exercise

All subjects in the intervention group continued to practice oral exercises at T2 and T3; in the control group, 28 and 29 subjects continued to practice oral exercises at T2 and T3. Chi-square analysis revealed no significant between-group differences in the number of subjects who continued oral exercises at each time point. However, the intervention group spent significantly more time practicing oral exercises than the control group. At T2, the intervention group exercised 261.0 min (95%CI: 137.5~384.5, *p* < 0.001) more than the control group, with a mean weekly exercise time of 383.3 (SD = 312.1) minutes in the intervention group and 122.3 (SD = 160.7) minutes in the control group. At T3, the intervention group exercised 217.1 min (95%CI: 107.4~326.7, *p* <0.001) more than the control group, with a mean weekly exercise time of 323.6 (SD = 291.2) minutes in the intervention group and 106.5 (SD = 104.7) minutes in the control group (Table 2). These results support the research hypothesis that the intervention group practiced more mouth-opening exercises than the control group.

### 3.3. Effects on Maximum Interincisal Opening

**Figure 1:**
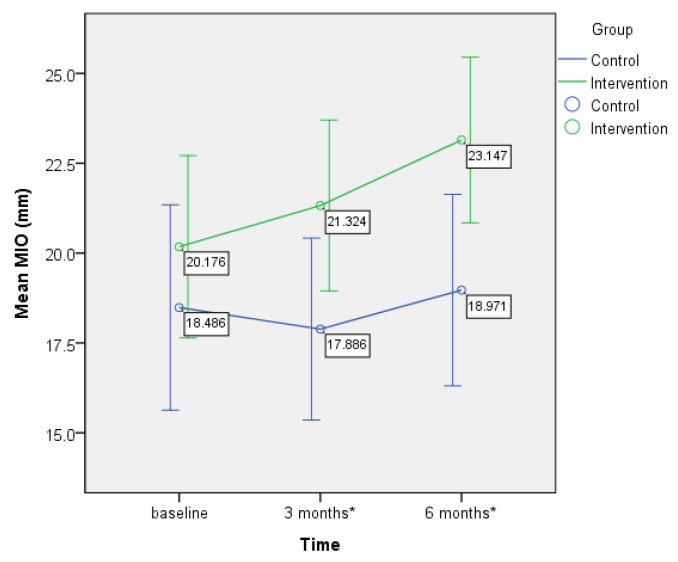
Changes in the distance of maximum interincisal opening overtime at the 1st month, 3rd month, and 6th month follow-up (error bars: 95%CI). * *p* < 0.05 for the between-group difference.

GEE modeling showed a statistically significant group-by-time interaction in MIO after controlling for subjects’ cancer location, cancer stage, free flap surgery, and adjuvant therapy. The change in MIO score from T1 to T3, as indicated by the regression slope, was 2.3 mm (95%CI: 0.2~4.5, *p* = 0.032) greater in the intervention group than in the control group (Table 3). These results support the research hypothesis that the intervention group experienced greater improvement in MIO over time compared to the control group. The mean MIO values of the subjects in the intervention group at T1, T2, and T3 were 20.2 (SD = 7.3), 21.3 (SD = 6.8), and 23.2 (SD = 6.6), respectively, and those of the subjects in the control group were 18.5 (SD = 8.3), 17.9 (SD = 7.4), and 18.98 (SD = 7.8), respectively. The independent *t*-test showed no significant between-group differences in MIO at T1. However, at T2, MIO was 3.4 mm (95%CI: 0.0~6.9, *p* = 0.048) higher in the intervention group than in the control group, with a Cohen’s d of 0.47 (medium effect size). At T3, MIO was 4.2 mm (95%CI: 0.7~7.6, *p* = 0.019) higher in the intervention group than in the control group (Table 2), with a Cohen’s d of 0.58 (medium effect size). Plotting as a linear graph, the MIO of the intervention group continued to increase from T1 to T3 (Figure 1). In contrast, the MIO of the control group remained essentially unchanged over time.

Taking MIO below 35 mm as the cut point for trismus, 33 (97.1%) subjects in the intervention group had trismus at all three time points, while 32 (91.4%), 33 (94.3%), and 34 (97.1%) subjects in the control group had trismus at T1, T2, and T3, respectively. Thus, there was a high proportion of subjects with trismus in all groups at one, three, and six months after radiotherapy, with no significant between-group differences.

### 3.4. Effects on Mandibular Function Impairment

Results of the GEE showed no significant intervention effects for mandibular function impairment, as the between-group and group-by-time interaction effects were insignificant (Table 3). The results also do not support the research hypothesis that, over time, the mandibular function impairment of the intervention group would improve more than that of the control group. The mean scores of mandibular function impairment of the intervention subjects at T1, T2, and T3 were 0.48 (SD = 0.24), 0.46 (SD = 0.21), and 0.44 (SD = 0.23), respectively, and those of the control subjects were 0.57 (SD = 0.25), 0.55 (SD = 0.21), and 0.51 (SD = 0.22), respectively. Results of independent *t*-tests showed no significant between-group differences in mandibular function impairment at all three data collection points (Table 2).

## 4. Discussion

Our findings support the effect of the proposed intervention on increasing mouth-opening exercise practice time in oral cancer patients treated with RT or CCRT. At the three-month follow-up, subjects in the intervention group spent 383.3 min per week doing mouth-opening exercises, significantly more than the 122.3 min per week in the control group. At the six-month follow-up, subjects in the intervention group still spent 323 min per week doing mouth-opening exercises, significantly more than the 106.5 min per week in the control group. Similar findings were reported in a previous study involving 60 postoperative oral cancer patients with a shorter follow-up period [20]. In that study, the intervention group (mouth-opening exercise education and telephone follow-up) exercised for 377 min per week compared to 299.67 min in the control group during the three-month follow-up. These findings support that intervention using personal training, educational media, and a follow-up phone call effectively increases adherence to mouth-opening exercises. However, the intervention group did 383.3 min of mouth-opening practice per week, which is a lot of time spent on one activity. Whether exercise time can be reduced without affecting results deserves further investigation.

Unlike the no-intervention effect reported in previous studies [22,23], our results support the effect of mouth-opening exercises for improving MIO after RT or CCRT in patients with oral cancer. The MIO of the subjects in the intervention group gradually improved over time, and the improvement was significantly greater than that in the control group. At the 3rd and 6th months of follow-up, the mean MIO of the intervention group was significantly higher than that of the control group by 3.4 mm and 4.2 mm, respectively. The discrepancy in the research outcome may be due to the fact that the intervention in our study also included facial muscle strengthening exercises such as cheek massage, jaw stretches, facial muscle exercise, tongue exercise, sucking, and chewing. Massaging the cheeks combined with jaw stretches relaxes the muscles and ligaments around the cheeks, promotes temporomandibular joint movement, and improves cheek control. Tongue moving and sucking exercises prevent or improve slurred speech and eating issues. Finally, chewing and intensive mouth-opening exercises strengthen the muscles of mastication and prevent trismus. Furthermore, our intervention’s exercise practice time and frequency are greater than those of these previous studies [22,23]. However, the effect size is smaller in our study compared to the results of 5.1 mm (95%CI: 0.6~8.6) at the three-month follow-up and 5.8 mm (95%CI: 4.9~6.8) at the six-month follow-up in the Wang et al. [13] meta-analysis. In addition, the effect sizes of our intervention were also smaller than what was reported in previous studies on jaw-mobilizing device-assisted exercises. Li et al. found (*n* = 40) that MIO improved by 14.2 mm (95%CI: 10.7~17.7) after 3 months of EZBbite-assisted exercises [24]. Tang et al. found (*n* = 40) a 6.00 mm (95%CI: 2.8~9.2) improvement in MIO after 3 months of TheraBite-assisted exercises [25]. Nevertheless, the jaw-mobilizing device-assisted exercises were painful, and the discomfort caused by the devices caused subjects to withdraw [26]. Our intervention is gentle and increases in difficulty incrementally to improve patient compliance and willingness to continue. However, more than 90% of subjects had trismus (MIO < 35 mm) at all three time points, highlighting the prevalence of this problem. Subsequently, there was no between-group difference in the number of subjects with trismus at any time. These findings suggest that although our intervention improved MIO, the improvement was small and did not reduce the incidence of trismus. The small beneficial effect might not be clinically relevant as it is smaller than 5 mm, the smallest detectable difference [27].

Furthermore, our results do not support the effect of the intervention on improving mandibular function impairment. This may be due to relatively small changes in MIO and may not produce significant changes in mandibular function impairment. Our subjects were mainly stage 4 (51.4%) and buccal mucosa cancer patients (65.7%). Most of these patients received broad-spectrum therapy, resulting in severe mandibular function impairment. In addition, we did not control factors affecting mandibular function impairment, such as stomatitis or pain intensity, which may limit the inferential power of the findings. Further investigation is needed to develop more effective interventions to treat trismus in this population.

Considering MIO declined rapidly from 1 to 9 months after RT [28] and treatment-related adverse effects or toxicities during adjuvant therapies, we started the oral exercise intervention after patients completed therapies. However, there are also potential advantages to initiating oral exercise during therapies, such as close monitoring and early prevention of MID reduction. Therefore, further investigation is needed to answer when it will be a better time to start oral exercise in this population.

Our study has some limitations. First, we recruited a convenience sample of oral cancer patients from a medical center in Taiwan. The characteristics of our subjects may differ from those of patients in other clinical settings. Findings cannot be generalized outside of the sample. Second, the findings were also biased by the large number of subjects who declined to participate in the study. Third, the inability to measure MID due to the absence of incisors as exclusion criteria may represent a bias in the study. This criterion determines the exclusion of a considerable number of elderly patients. Fourth, the study was based on a quasi-experimental design. Instead of recruiting subjects for both study groups simultaneously, we recruited the control group before the study group. This could pose a threat of historical bias or selection bias. Furthermore, in the absence of randomization, it is difficult to exclude baseline differences between the two study groups. Although no significant between-group differences were found in baseline values of subject characteristics and outcome values, only random assignment could ensure true group equivalence. Fifth, we could not blind our subjects from group assignments and, therefore, could not rule out the Hawthorne effect. Sixth, our subjects practiced speaking practice at home without supervision or objective monitoring. Although subjects kept exercise diaries, the correctness of the diary entries could not be verified. Seventh, the sample size required for our study was estimated from the primary outcome (MIO), which may not have enough statistical power to detect changes in the secondary outcomes (mandibular function impairment). Eighth, this study only focused on the effect of oral exercise on increasing the maximum incision opening. If patient-reported outcomes could also be incorporated into the study design, it would give us insight into how patients felt about the intervention and whether they improved their overall quality of life. Lastly, oral cancer patients’ MID can decline rapidly for up to one year after radiation therapy. Given the potential for further adverse development [29], the 6-month follow-up in the current study may be too short. A longer follow-up may help to understand whether the study results would hold after a year or two.

## 5. Conclusions

Trismus is a severe complication of oral cancer treatment. Preventing and reducing trismus is essential for improving patient outcomes and quality of life. The study findings showed that oral exercise intervention improved subjects’ exercise adherence and MIO. Personal training, oral exercise tutorial videos, educational brochures, and phone follow-ups can increase oral exercise engagement among patients with oral cancer. Although the intervention did not reduce the number of subjects with trismus nor did it improve mandibular function impairment, six months of oral exercise moderately improved MIO. The results of this study provide preliminary support for the benefits of oral exercise interventions for improving mouth opening in patients undergoing radiotherapy for oral cancer. However, additional randomized controlled trials with more extensive or diverse samples are needed to examine interventions’ effectiveness, design, and timing. Although more research is needed, oral exercise intervention is recommended for patients with oral cancer because it is gentle, safe, and potentially beneficial.

## Figures and Tables

**Table 1:** Demographics and baseline equilibrium.

Variables	Total (n = 69)		Intervention (*n* = 34)		Control (*n* = 35)		Between-Group Comparisons	
	Mean	SD	Mean	SD	Mean	SD	*t*	*p*
Age	50.3	7.4	50.2	7.4	50.3	7.6	0.04	0.965
	n	%	n	%	n	%	X^2^	*p*
Gender							0.32	1.00 ^a^
Male	66	95.7	33	97.1	33	94.3		
Female	3	4.3	1	2.9	2	5.7		
Education							3.40	0.335
Elementary	15	21.7	6	17.6	9	25.7		
Middle	20	29	10	29.4	10	28.6		
High	28	40.6	13	38.2	15	42.9		
College	6	8.7	5	14.7	1	2.9		
Marital status							3.56	0.093
Single	17	24.6	5	14.7	12	34.3		
Married	52	75.4	29	85.3	23	65.7		
Working status							1.07	0.437 ^a^
No	22	32.4	13	38.2	9	26.5		
Yes	46	67.6	21	61.8	25	73.5		
Cancer locations							0.86	0.835
Buccal mucosa	43	62.3	20	58.8	23	65.7		
Hard palate	4	5.8	2	5.9	2	5.7		
Gingiva	16	23.2	8	23.5	8	22.9		
Retromolar trigone	6	8.7	4	11.8	2	5.7		
Cancer stage							0.48	0.924
I	3	4.4	1	3.0	2	5.7		
II	24	35.3	12	36.4	12	34.3		
III	5	7.4	2	6.1	3	8.6		
IV	36	52.9	18	54.5	18	51.4		
Free flap surgery							0.68	1.09
Yes	65	94.2	32	94.1	33	94.3		
No	4	5.8	2	5.9	2	5.7		
Adjuvant therapy							0.03	1.00 ^a^
Radiotherapy	21	30.4	10	29.4	11	31.4		
CCRT ^b^	48	69.6	24	70.6	24	68.6		

^a^ Fisher’s exact test. ^b^ concurrent chemoradiation therapy.

**Table 2:** Between-group comparisons on the exercise time, maximum interincisal opening, and mandibular function impairment at three time points.

Variables	Time	Intervention			Control			Between-Group Comparisons		
		*n*	Mean	SD	*n*	Mean	SD	Mean Difference	95%CI	*p*
Exercise time	T2	34	383.3	312.1	28	122.3	160.7	261.0	137.5~384.5	<0.001 ***
	T3	34	323.6	291.2	29	106.5	104.7	217.1	107.4~326.7	<0.001 ***
Maximum interincisal opening	T1	34	20.2	7.3	35	18.5	8.3	1.7	−2.1~5.5	0.372
	T2	34	21.3	6.8	35	17.9	7.4	3.4	0.0~6.9	0.048 *
	T3	34	23.2	6.6	35	19.0	7.8	4.2	0.7~7.6	0.019 *
Mandibular function impairment	T1	34	0.48	0.24	35	0.57	0.25	−0.09	−0.21~0.03	0.132
	T2	34	0.46	0.21	34	0.55	0.21	−0.10	−0.20~0.01	0.066
	T3	34	0.44	0.23	34	0.51	0.22	−0.07	−0.18~0.04	0.198

T1, baseline; T2, 3 months; T3, 6 months; * *p* < 0.05; *** *p* < 0.001

**Table 3:** Parameters of the generalized linear model for the effect of oral exercise on maximum interincisal opening and mandibular function impairment.

Variables ^a^	ß	SE	95%CI	*X* ^2^	*p*
Maximum interincisal opening					
Group (intervention) ^b^	1.49	1.78	−1.99~4.98	0.70	0.401
Time (6 months) ^c^	0.49	0.76	−1.00~1.97	0.41	0.522
Time (3 months) ^c^	−0.60	0.76	−2.09~0.89	0.63	0.429
Group (intervention) × time (6 months) ^d^	2.33	1.09	0.20~4.47	4.59	0.032 *
Group (intervention) × time (3 months) ^d^	1.57	1.09	−0.56~3.70	2.08	0.149
Retromolar trigone cancer ^e^	4.71	3.13	−1.43~10.85	2.26	0.133
Gingiva cancer ^e^	0.00	2.10	−4.11~4.12	0.00	0.999
Hard palate cancer ^e^	−3.99	3.63	−11.10~3.12	1.21	0.272
Cancer stage I ^f^	−0.24	4.31	−8.68~8.21	0.00	0.957
Cancer stage II ^f^	1.20	1.92	−2.58~4.97	0.39	0.534
Cancer stage III ^f^	3.65	3.35	−2.91~10.21	1.19	0.275
Free flap surgery ^g^	1.14	3.75	−6.21~8.49	0.09	0.761
Concurrent chemoradiotherapy ^h^	2.91	1.91	−0.82~6.64	2.33	0.127
Mandibular function impairment					
Group (intervention) ^b^	−0.10	0.05	−0.21~0.00	3.54	0.060
Time (6 months) ^c^	−0.07	0.030	−0.12~(−0.01)	5.05	0.025 *
Time (3 months) ^c^	−0.02	0.030	−0.08~0.04	0.55	0.464
Group (intervention) × time (6 months) ^d^	0.05	0.04	−0.03~0.13	1.37	0.241
Group (intervention) × time (3 months) ^d^	−0.00	0.04	−0.09~0.08	0.01	0.938
Retromolar trigone cancer ^e^	−0.01	0.09	−0.19~0.17	0.02	0.900
Gingiva cancer ^e^	0.05	0.06	−0.07~0.17	0.64	0.424
Hard palate cancer ^e^	−0.05	0.11	−0.25~0.16	0.19	0.660
Cancer stage I ^f^	−0.03	0.13	−0.28~0.21	0.074	0.786
Cancer stage II ^f^	−0.11	0.06	−0.22~0.00	3.826	0.050
Cancer stage III ^f^	−0.17	0.10	−0.36~0.02	3.002	0.083
Reconstruction surgery ^g^	0.07	0.11	−0.14~0.29	0.45	0.503
Concurrent chemoradiotherapy ^h^	−0.07	0.06	−0.18~0.04	1.57	0.210

^a^ Using generalized estimating equation for repeated measurements and an exchangeable correlation structure. ^b^ Reference group: control group. ^c^ Reference group: time (baseline). ^d^ Reference group: group (control) × time (baseline). ^e^ Reference group: buccal mucosa. ^f^ Reference group: cancer stage IV. ^g^ Reference group: without reconstruction surgery. ^h^ Reference group: radiotherapy. * *p* < 0.05.

## Data Availability

Data will be available from the corresponding author upon reasonable request.

## Appendix A

**Table A1:** Details of the oral exercise intervention.

Components	Descriptions of the Movements	Repeat
1. Cheek massage	Gently massage the cheeks and surrounding tissues with the index and middle fingers for about 1 min.	-
2. Jaw stretch	Open the mouth and move the chin to the left, right, and forward, holding each action for 3 s. Then, open the mouth as wide as possible and hold for 10 s.	Ten times
3. Facial muscle movement	Raise the eyebrows, wrinkle the nose, pout the mouth, puff the cheeks, and smile.	Ten times
4. Tongue movement	Roll the tongue forward, backward, up, down, left, and right, and press it against each cheek. Then, extend the tongue and make a circular motion around the lips.	Ten times
5. Sucking	Pout the mouth and make a sucking motion.	Ten times
6. Chewing	Clench the upper and lower jaws tightly.	Ten times
7. Intensive mouth opening	Insert a stack of depressors (as many as possible) into the mouth between the upper and lower incisors. Once inserted, push one more depressor into the stack, hold for 10–15 min, and gradually increase the number of depressors over time.	-
